# Mitigation of Benzene-Induced Haematotoxicity in Sprague Dawley Rats through Plant-Extract-Loaded Silica Nanobeads

**DOI:** 10.3390/toxics11100865

**Published:** 2023-10-17

**Authors:** Touseef Rehan, Anum Tahir, Aneesa Sultan, Khulood Fahad Alabbosh, Shahid Waseem, Mazhar Ul-Islam, Khalid Ali Khan, Essam H. Ibrahim, Muhammad Wajid Ullah, Nasrullah Shah

**Affiliations:** 1 Department of Biochemistry, Women University Mardan, Mardan 23200, Pakistan; 2 Department of Biochemistry, Quaid-i-Azam University, Islamabad 45320, Pakistan; 3 Department of Biology, College of Science, University of Hail, Hail 2440, Saudi Arabia; 4 Department of Chemical Engineering, College of Engineering, Dhofar University, Salalah 211, Oman; 5 Unit of Bee Research and Honey Production, Research Center for Advanced Materials Science (RCAMS), Applied College, King Khalid University, Abha 61413, Saudi Arabia; 6 Biology Department, Faculty of Science, Research Center for Advanced Materials Science (RCAMS), King Khalid University, Abha 61413, Saudi Arabia; 7 Blood Products Quality Control and Research Department, National Organization for Research and Control of Biologicals, Cairo 12611, Egypt; 8 Biofuels Institute, School of the Environmental and Safety Engineering, Jiangsu University, Zhenjiang 212013, China; 9 Department of Chemistry, Abdul Wali Khan University Mardan, Mardan 23200, Pakistan

**Keywords:** Lamiaceae plant extracts, mesoporous silica nanoparticles, *in vivo* study, targeted drug delivery

## Abstract

Benzene, a potent carcinogen, is known to cause acute myeloid leukaemia. While chemotherapy is commonly used for cancer treatment, its side effects have prompted scientists to explore natural products that can mitigate the haematotoxic effects induced by chemicals. One area of interest is nano-theragnostics, which aims to enhance the therapeutic potential of natural products. This study aimed to enhance the effects of methanolic extracts from *Ocimum basilicum*, *Rosemarinus officinalis*, and *Thymus vulgaris* by loading them onto silica nanobeads (SNBs) for targeted delivery to mitigate the benzene-induced haematotoxic effects. The SNBs, 48 nm in diameter, were prepared using a chemical method and were then loaded with the plant extracts. The plant-extract-loaded SNBs were then coated with carboxymethyl cellulose (CMC). The modified SNBs were characterized using various techniques such as scanning electron microscopy (SEM), X-ray diffraction (XRD), UV–visible spectroscopy, and Fourier transform infrared (FTIR) spectroscopy. The developed plant-extract-loaded and CMC-modified SNBs were administered intravenously to benzene-exposed rats, and haematological and histopathological profiling was conducted. Rats exposed to benzene showed increased liver and spleen weight, which was mitigated by the plant-extract-loaded SNBs. The differential white blood cell (WBC) count was higher in rats with benzene-induced haematotoxicity, but this count decreased significantly in rats treated with plant-extract-loaded SNBs. Additionally, blast cells observed in benzene-exposed rats were not found in rats treated with plant-extract-loaded SNBs. The SNBs facilitated targeted drug delivery of the three selected medicinal herbs at low doses. These results suggest that SNBs have promising potential as targeted drug delivery agents to mitigate haematotoxic effects induced by benzene in rats.

## 1. Introduction

Benzene, an aromatic compound, has been linked to human cancers such as leukaemia. When benzene is metabolically converted, it produces phenol, hydroquinone, and catechol, which are responsible for causing cytogenetic changes and affecting the cell cycle [1]. These compounds can lead to genetic damage, including structural and chromosomal aberrations in the blood and bone marrow of exposed individuals. Benzene-induced leukaemia, involving oxidative DNA damage, is often seen in cases of acute myeloid leukaemia (AML), causing myelotoxicity and genotoxicity [2]. In AML, there is an increase in myeloid cells in the bone marrow and blood. Immature blast cells are produced in the bone marrow, which do not fully mature and are unable to effectively defend against diseases. These immature cells grow rapidly, decreasing the number of mature white blood cells (WBCs) and leading to granulocytopenia, thrombocytopenia, and anaemia, either with or without leukocytosis [3]. The haematotoxicological effects of benzene are well-known, particularly in environments such as petrochemical industries, refineries, and mechanical workshops, where exposure to benzene and its harmful components is common. This poses a significant risk to the population, with higher chances of developing haematotoxicity. These biohazardous pollutants have gained attention from the public and scientists worldwide [4,5]. 

Nanobiotechnology is an emerging field that utilizes nano-sized materials (ranging from 10 to 100 nm) in various biological applications [6]. Different types of nanoparticles have been developed and characterized for their role in biomedical sciences [7,8]. One of the biggest challenges in disease treatment is delivering chemotherapeutic drugs to the specific pathological site [9]. To address this issue, a controlled drug delivery system (DDS) [10] can be designed to enhance the properties of conventional (free) drugs, ensuring their proper distribution and release at the target site [11]. Nanoparticles aid in delivering drugs to pathogenic sites while minimizing loss in blood circulation and minimizing damage to normal cells, making targeted drug delivery possible [12]. Silica, due to its cheap, thermally stable, harmless, and chemically inert nature, has gained significant attention [13]. Silica nanoparticles, like other inorganic nanoparticles, have shown promising potential in biomedical research as an innovative drug delivery system [14,15]. They possess various remarkable features such as easy manufacturing, tuneable pore size, diversity in functionalization, and the ability to carry multiple drugs and proteins within their pores [16,17,18]. A toxicity study revealed that mice exposed to silica nanoparticles continuously for 14 days through intravenous administration at doses of 20, 40, and 80 mg/kg experienced no deaths. The clearance of 50 nm particles was observed through urine and bile, while 100 nm and 200 nm particles were detected in urine and faecal analysis [13]. The surface functionalization of nanoparticles plays a crucial role in determining their fate and lifespan in the blood, as well as targeted drug delivery. To prolong drug release, resins were coated with ethyl cellulose or carboxymethyl cellulose (CMC), which delayed drug elution at the target site [19]. The immobilization of ethyl cellulose improves the aqueous stability and photostability of the drug [9]. 

Natural products exert their effects through the detoxification of substances, using enzymes that have antioxidant activity, as well as their anti-inflammatory potential and their ability to inhibit the cell cycle and induce cell death [20]. Medicinal plants are now being frequently studied for their potential in treating cancers and various ailments [21,22]. The Lamiaceae family, which includes the genus *Ocimum*, is a major producer of essential oils [23]. Basil, rosemary, and thyme, which are all part of this family, contain phenolic compounds that have shown antitumor, antimicrobial, and antioxidant properties [24]. These phenolic compounds have redox properties, which help neutralize free radicals and decompose peroxides [25]. Methanolic extracts of basil have demonstrated significant activity against benzene-induced haematotoxicity in mice [26]. Plants from the Lamiaceae family are known for their biological activities as they contain various active compounds with the potential to reduce the harmful effects of the environment and drugs [27]. The antioxidant activity of basil (*Ocimum basilicum*) extracts is due to the ability of phenolic acids to absorb and neutralize free radicals, quench singlet and triplet oxygen, and decompose peroxides [28], thus showing antimicrobial, antioxidant, and antitumor activities [29]. Rosemary (*Rosmarinus officinalis*), a commonly used plant in medicine and cosmetics, has various *in vitro* and *in vivo* bioactivities, including potent antioxidant potential and anti-inflammatory, antimutagenic, and antimetastatic effects [18,30]. Rosemary extract has been shown to inhibit MAPK and ERK pathways and antagonize the expression of activator protein-1-dependent COX-2 [31]. Thyme (*Thymus vulgaris*), a perennial herb found in Europe and Asia, is traditionally used in the treatment of bronchopulmonary and gastroenteric disorders [32]. Thyme contains two major compounds, thymol and carvacrol, which make up 75% of its total volatiles, as well as trace amounts of linalool, 1,8-cineole, α-terpineol, and borneol [33]. The essential oil of thyme has been found to have protective effects against Aflatoxin B, a well-known hepatocarcinogen [34]. *In vivo* and *in vitro* studies have shown that thymol has cytoprotective effects against radiation-induced damage. Thymol and carvacrol have also been reported to protect cells from DNA damage induced by H_2_O_2_ in the colon, hepatoma, and leukemic cell lines [35].

There is currently limited information available on the antitoxic effects of medicinal plant extracts, particularly those from the Lamiaceae plants. Therefore, this study aimed to investigate whether the application of methanolic leaf extracts of selected plants from the Lamiaceae family could help reduce the haematotoxic effects caused by benzene injection in rats. The synthesized silica nanobeads were characterized using various techniques and loaded with plant extracts. Thereafter, these were administered intravenously to the rats. Haematological and histopathological profiling, as well as observation of blast cells, were carried out. The findings of this study suggest that silica nanobeads have potential as targeted drug delivery agents to mitigate the haematotoxic effects of benzene.

## 2. Materials and Methods

### 2.1. Collection and Preparation of Plant Material

Plant material from *O. basilicum*, *T. vulgaris*, and *R. officinalis* was obtained from the clonal repository of the National Agriculture Research Center (NARC), Islamabad, Pakistan. The leaves were collected from plants grown in glasshouse conditions and shadow dried. The dried leaves were ground to create a coarse powder. This powder was soaked in methanol for 24 h under continuous shaking. Afterwards, the mixture was filtered using Whatman filter paper, and a filtrate was obtained. The extraction solvent was completely removed via distillation processes using a rotary evaporator (Bibby RE200B, Scientific, Col-Parmer-Antylia Sc. Stone, UK). The prepared extract was then used for further experiments. 

### 2.2. Synthesis of Silica Nanobeads

Silica beads (SNBs) were synthesized using a chemical method. First, a 0.25 wt.% solution of cetyltrimethylammonium bromide (CTAB) was prepared by mixing it for 1 h at 80 °C. Then, 5 mL of silica precursor tetraethyl orthosilicate (TEOS) was added drop-wise to the 0.25 wt.% CTAB solution. After 2 min of incubation at room temperature, the mixture became turbid, indicating hydrolysis of silicate. The mixture was stirred for 2 h. In parallel, a second solution containing 0.07 M NaOH solution was heated at 80 °C under magnetic stirring. Afterwards, the two solutions were mixed and stirred overnight. The solution was then left to settle until the particles completely settled. The obtained filtrate was then calcinated. For this purpose, the filtrate was oven-dried for 24 h each at 60 °C and 100 °C. After complete drying, the product was collected and made into fine powder form.

### 2.3. Characterization

The optical absorption spectra of uncoated SNBs, as well as those coated with plant extracts (*O. basilicum*, *T. vulgaris*, and *R. officinalis*) CMC, were measured using a UV–Vis spectrophotometer (Lambda 25 UV–vis, Perkin Elmer, Waltham, MA, USA) in the wavelength range of 200–800 nm. For the LC-MS study, an ion trap LC/MS system (Serial No. DE6220680) consisting of a liquid chromatograph (1200 Series, Agilent Tech, Santa Clara, CA, USA), a vacuum degasser, autosampler, a 4.6 × 12.5 mm column (XDB-C18, Agilent Tech, Santa Clara, CA, USA), and a diode array detector was used. MS data was obtained using an MS detector with an electrospray ion source and time of flight analyser (Agilent Tech, CA, USA). Gradient elution was performed with an initial mobile phase composition of 10% CH_3_CN organic solvent in 90% double-distilled H_2_O aqueous solvent. The composition was varied during the analysis as follows: 10% (0 min), 10% (15 min), 40% (40 min), 80% (50 min), and 10% (60 min). The flow rate was set at 0.5 mL/min, and the column temperature was maintained at 25 °C. An injection of 5 µL was used. The MS spectra were recorded in the range of 100–1000 *m*/*z* with a scan time of 0.5 s. XRD analysis of the SNBs was conducted using an X-ray spectrophotometer (Bruker D8 Advance, Karlsruhe, Germany) with Cu-Kα radiation (λ = 1.54 Å), operated at 40 kV and 30 mA. The average particle size was determined using the Debye–Scherrer equation (D = 0.9 λ/*βcosθ*), based on full-width and half-maximum (FWHM) values. The microscopic features of SNBs were observed using a scanning electron microscope (SEM, JEOL JSM-5910, JEOL, Tokyo, Japan). The specimens were observed without any metal coating in order to observe the genuine pore structures on the external surface. The particle size was determined using the ImageJ software (Version 1.53t) by randomly selecting 100 particles from the SEM images. For FTIR analysis, a 10 mg dried powder of each plant extract was encapsulated in a 100 mg pellet of KBr to prepare translucent sample discs. The powdered samples were then loaded in an FTIR spectrophotometer (Bruker, Tensor 27) with a scan range of 400 to 4000 cm^−1^ with a resolution of 4 cm^−1^.

### 2.4. Drug Loading on SNBs

After synthesizing SNBs, the methanolic extracts of *O. basilicum, T. vulgaris*, and *R. officinalis* were applied to them. The plant extract was dissolved in ethanol and sonicated until the drug was fully dissolved. Then, the SNPs were combined with the drug solution and left to stir overnight. The solution was then air-dried and mashed, resulting in a powder form.

### 2.5. CMC Coating on Plant-Extract-Loaded SNBs

To coat CMC on plant-extract-loaded SNBs, 0.009 g CMC was dissolved in 5 mL of dimethyl sulfoxide. Additionally, another solution was prepared by dissolving 0.008 g of plant-extract-loaded SNBs in 4.1 mL of dimethyl sulfoxide. Both solutions were mixed while stirring for 2 h and then sealed to prevent contamination.

### 2.6. In Vivo Studies

Male Sprague Dawley rats aged 6–8 weeks were procured from the National Institute of Health (NIH) laboratories and housed in an animal facility at Quaid-i-Azam University, Islamabad, Pakistan. The rats were maintained in accordance with the Helsinki Declaration and FELASA recommendations, ensuring proper nutrition, filtered water, and controlled room temperature. All animal handling procedures strictly adhered to the guidelines outlined by the Ethical Committee of Quaid-i-Azam University (Approval ID: BEC-FBS-QAU-09, Letter No. DFBS/17, Dated: 10 April 2017).

### 2.7. Animal Study

Thirty-five rats were divided into five groups (G1, G2, G3, G4, and G5), each consisting of seven rats. Group G1 served as the control and included healthy rats. Group G2 rats were treated with benzene to induce haematotoxicity, while Group G3 rats were treated with SNBs coated with *O. basilicum* extract. Group G4 received treatment with an extract of *R. officinalis*, and Group G5 included rats treated with an extract of *T. vulgaris*. The weights of rats were recorded before and after treatment, and they were regularly examined throughout the experiment.

### 2.8. Induction of Benzene Haematotoxicity in Sprague Dawley Rats

To induce haematotoxicity, a 0.2 mL solution was prepared containing benzene, injection water, and isopropanol in a ratio of 1.5:1.5:2. The solution was then injected intravenously on alternative days for three weeks. A separate group of healthy control rats were given 0.9 wt.% normal saline injections intravenously for three consecutive weeks. After treatment, the animals were kept for one more week and then euthanized and dissected. The animals were kept under standard laboratory conditions, including controlled temperature and relative humidity. All experiments were conducted in triplicates.

### 2.9. Drug Administration 

Rats were given three types of nanomedicine treatments for two weeks prior to injection of benzene *via* gastric gavage. The final form of SNBs (extracts + CMC-coating) was administered to the rats at a dosage of 0.1 mL/day/rat on alternate days. After the initial two weeks of treatment, the rats were injected with benzene along with continued nanomedicine treatments on alternate days for three consecutive weeks. After 3 weeks, the benzene injections were stopped, but the rats continued to receive the prepared nanomedicine treatment for an additional week. Throughout the experiment, the rats were observed for behavioural changes, such as fur changes, increased urination, irregular heartbeat, body weight changes, and mortality, to analyse subacute toxicity. The benzene-treated rats exhibited decreased mobility and weakness after completing the benzene injection treatment. After a total of 28 days (3 weeks of benzene solution treatment and 1 week of post-treatment with nanomedicine), rats were dissected, and blood samples were collected *via* cardiac puncture using disposable sterile 3-5 mL syringes in EDTA tubes. The weights of four organs (liver, heart, kidney, and spleen) were recorded and were preserved for further histopathology examination. The relative organ weight of rats was determined as follows.

$$Relative\;organ\;weight=\frac{Absolute\;organ\;weight\;(g)}{Body\;weight\;of\;rat\;on\;sacrifice\;day}$$

### 2.10. Blood Profiling of Rats

Rats from each group (G1–G5) were anaesthetized, and their abdomen was opened by making an incision in the middle. Blood was collected directly from the heart and transferred to EDTA tubes. Slides were prepared by making smears of the blood and allowing them to dry. The cells on the slides were then fixed in absolute methanol for 10 min and stained with Giemsa dye (MAKE, Country) diluted to a 10× concentration in PBS. Finally, the slides were examined under a light microscope (IRMECO, Lutjensee, Germany) to analyse the morphology of the blood cells and perform a differential count. 

### 2.11. Bone Marrow Staining

The bone marrow of the rats was collected from the femurs and tibiae by flushing the shaft with PBS using a syringe with a 26G needle. Gentle pipetting was performed to avoid disaggregation. The cells were then centrifuged at 200× *g* for 10 min, and the supernatant was removed. The resulting cell pellet was suspended in PBS. The bone marrow cells were fixed on a slide stained with Giemsa dye. Morphological studies were conducted under a light microscope (IRMECO, X100).

## 3. Results

### 3.1. UV–Vis Spectroscopy of Plant-Extract-Loaded SNBs and UV/LC-MS of Plant Extracts

The absorbance spectra of naked SNBs were measured from 200–800 nm, and no absorbance was observed in the visible region for silica particles. The UV–Vis spectra of crude extracts from *O. basilicum, T. vulgaris*, and *R. officinalis* were also measured in the same range. Results showed absorption peaks for *O. basilicum* at approximately 290 nm, 410 nm, and 670 nm. *T. vulgaris* crude extract displayed absorbance at 420 nm and 680 nm. *R. officinalis* crude extract exhibited absorbance at approximately 670 nm. The absorption spectra of the SNBs loaded with three types of medicinal plant extracts were recorded to confirm the successful loading of plant extracts onto the SNBs. Results showed that the peaks observed in the loaded forms matched those found in the extracts of *O. basilicum, T. vulgaris*, and *R. officinalis*, indicating successful loading of the plant extracts onto the nanobeads.

Table 1 presents the UV range and LC-MS results of selected fractions of the methanolic extracts of *O. basilicum, R. officinalis*, and *T. vulgaris*. MS spectra of different fractions of methanolic extracts and their corresponding LC-DAD chromatograms are shown in Figures S1 and S2, respectively. The UV spectroscopy study revealed that the major compounds present in the fractions exhibited maximum absorption in the range of 230–330 nm. LC-MS data was interpreted by comparing the spectra with the MS data bank and following the fragmentation pattern method. The major compounds identified were epicatechin derivatives, caffeic acid-3-glucoside dimers, ester derivatives of D-Alanine, ester derivatives of cinnamic acid, derivatives of Kaempferol (flavonoid), carboxylic acid derivatives, ester derivatives of succinic acid, and ester derivatives of cinnamic acid. The compounds found in the two selected fractions were similar to a great extent, with minor differences. These results are consistent with the FTIR results of the respective fractions, which matched the functional groups correlated to the compounds identified using LC-MS.

### 3.2. Structural Morphology of SNBs

**Figure 1:**
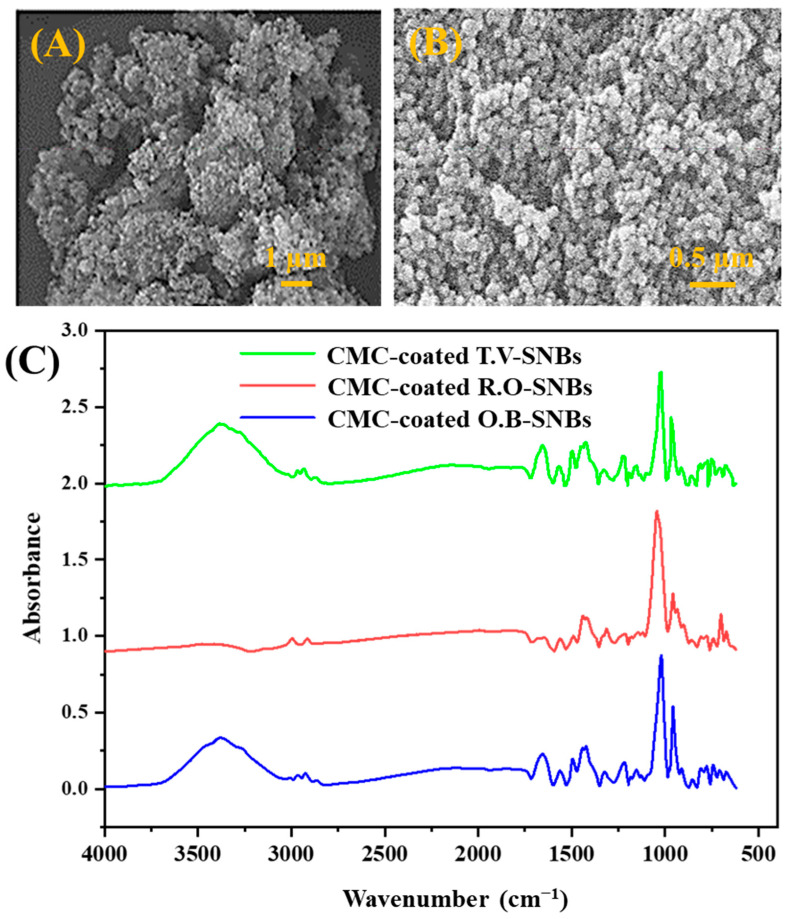
SEM micrographs of SNBs at (**A**) lower magnification and (**B**) higher magnification. (**C**) FTIR spectra of different CMC-coated plant-extract-loaded SNBs.

The SEM was used to observe the microscopic features of the SNBs. To study the surface morphology and shape of the beads, the SNBs were observed without any coating. Figure 1A,B show SEM images captured at different magnifications. The SEM observation revealed that the prepared SNBs were predominantly round shaped, although some agglomeration was observed.

### 3.3. Chemical Structure Analysis of SNBs

The FTIR spectra were recorded for various samples, including SNBs, extracts of *O. basilicum, T. vulgaris*, and *R. officinalis*, plant-extract-loaded SNBs, CMC-coated SNBs, and final formed product (plant-extracts-loaded SNBs encapsulated with CMC) (Figure 1C). The absorption band at 3370 cm^−1^ corresponds to the stretching vibration of O-H bonds, which are present in both the organic compounds and Si-OH groups of SNBs. Peaks at 3000–3050 cm^−1^ indicate the sp^2^ C-H stretching in aromatic compounds. The peaks in the range of 2900–2980 cm^−1^ and 2860–2890 cm^−1^ are attributed to the stretching vibrations of sp^3^ C–H bonds. The dominant peak at 1660 cm^−1^ clearly represents carbonyl groups in the organic molecules. A recessive band at 2150 cm^−1^ may be attributed to the C≡C stretching vibrations range, while a sharp peak at 1580 cm^−1^ arises from aromatic C=C bonds as this range approximates the C=C vibrations of aromatic compounds. The absorption peaks at 1430 cm^−1^ and 1500 cm^−1^, along with a very sharp and dominant peak at 1030–1050 cm^−1^, indicate the presence of an aromatic group C-C stretch absorption band. This is followed by a peak at 950 cm^−1^, which could be due to the C–O stretching vibration of an ester group or secondary alcohol.

### 3.4. XRD Analysis of Particle Size of SNBs

**Figure 2:**
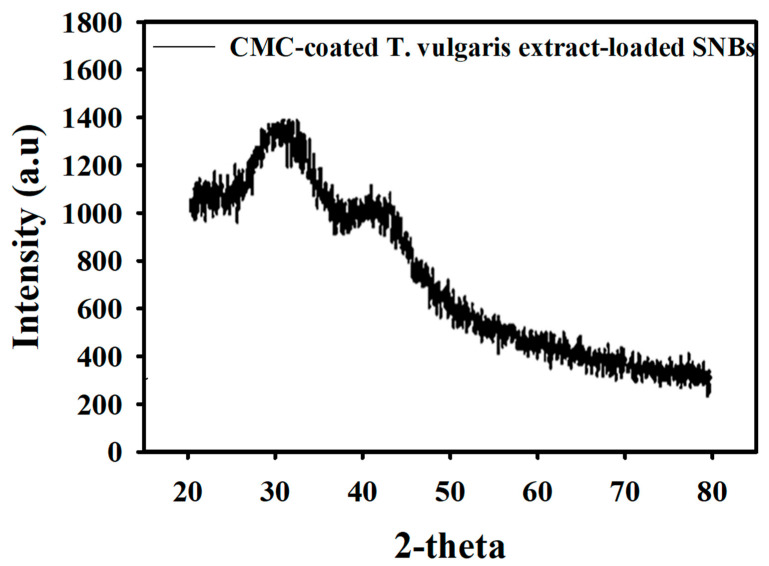
XRD spectrum of the CMC-coated and *T. vulgaris*-extract-loaded SNBs.

**Figure 3:**
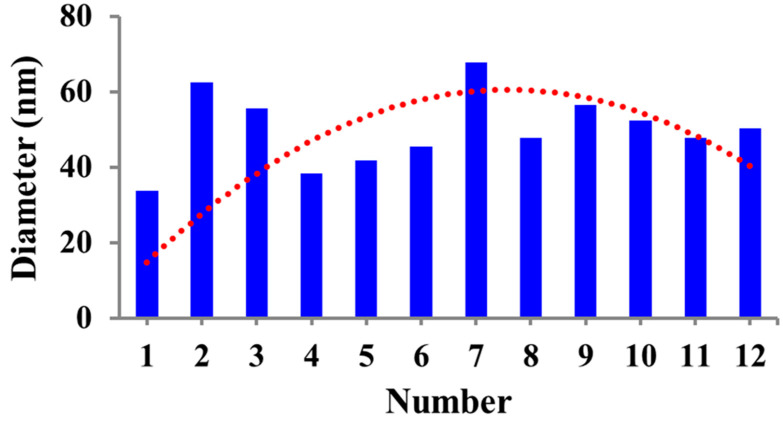
Particle size distribution of SNBs calculated through ImageJ software from SEM micrographs.

The XRD pattern of the SNBs is displayed in Figure 2, revealing a diffraction peak at 2θ = 31. By applying the Debye–Scherrer equation, the size of the SNBs was determined based on their FWHM. The calculations indicated that the SNBs have a diameter of approximately 47.94 nm. Furthermore, the average size of the beads, as calculated using ImageJ software, closely matched the average diameter obtained from the Scherrer equation, as depicted in Figure 3.

### 3.5. Relative Organ Weight Measurement

**Figure 4:**
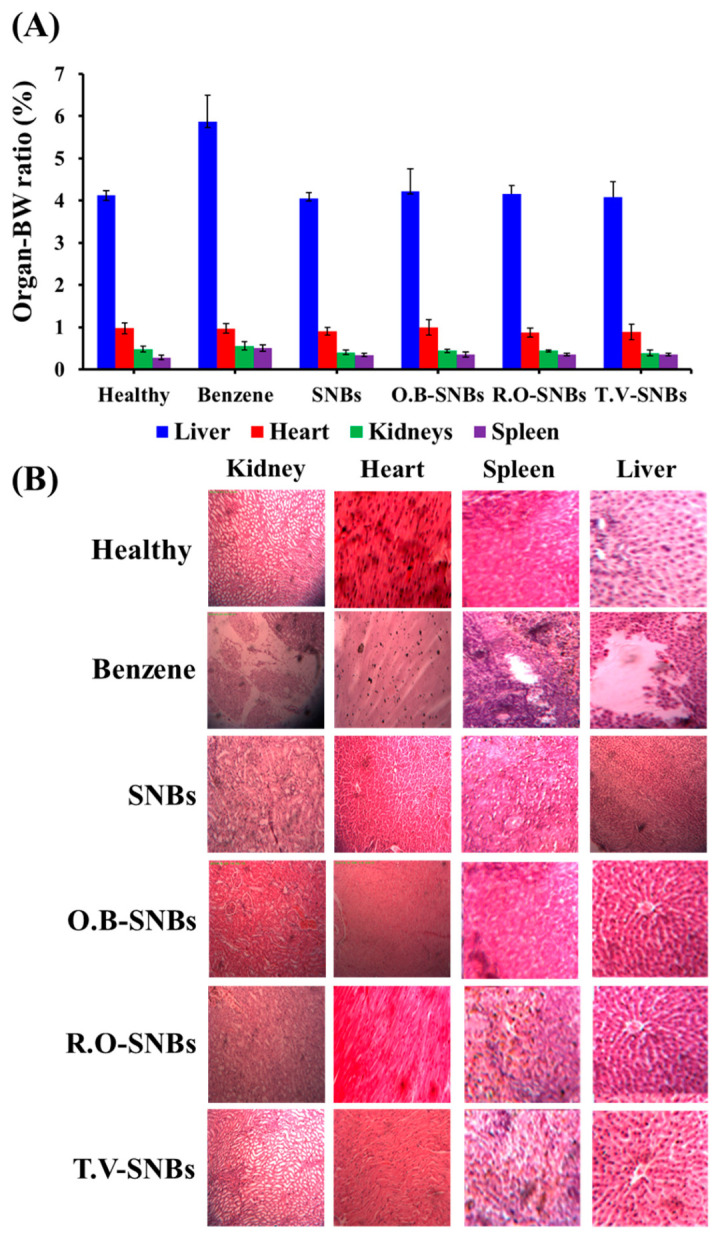
(**A**) Data of relative organ–body weight ratio of healthy rats, those treated with benzene, those treated with only SNBs, those treated with *O. basilicum* plant extract loaded on SNBs (O.B-SNBs), those treated with *R. officinalis* plant extract loaded on SNBs (R.O-SNBs), and those treated with *T. vulgaris* plant extract loaded on SNBs (T.V-SNBs). (**B**) Histopathological examination of the kidney, heart, spleen, and liver of healthy rats, those treated with treated with benzene, those treated with only SNBs, those treated with *O. basilicum* plant extract loaded on SNBs (O.B-SNBs), those treated with *R. officinalis* plant extract loaded on SNBs (R.O-SNBs), and those treated with *T. vulgaris* plant extract loaded on SNBs (T.V-SNBs).

The body weight of the rats was measured three times: before the start of the experiment, after injection of benzene, and at the time of dissection after treatment with nanomedicines. The average organ body weight ratio was then calculated for all five groups: control (G1), rats injected with benzene (G2), rats treated with *O. basilicum*, *T. vulgaris*, and *R. officinalis* extract-loaded SNBs (G3–G5). The organ–body weight ratio of the liver and spleen was found to be higher in rats injected with benzene compared to healthy rats (control). However, rats injected with benzene but treated with SNBs loaded with the three selected medicinal plant extracts had normal weights of these organs, similar to those of rats without leukaemia. No significant changes were observed in the relative organ–body weight ratio for other organs. Notably, the weight of the liver was significantly decreased in rats injected with benzene and treated with SNBs loaded with *T. vulgaris* (Figure 4A). The corresponding histopathological micrographs of kidney, heart, spleen, and liver are shown in Figure 4B.

### 3.6. Differential Counting of Blood Cells

**Figure 5:**
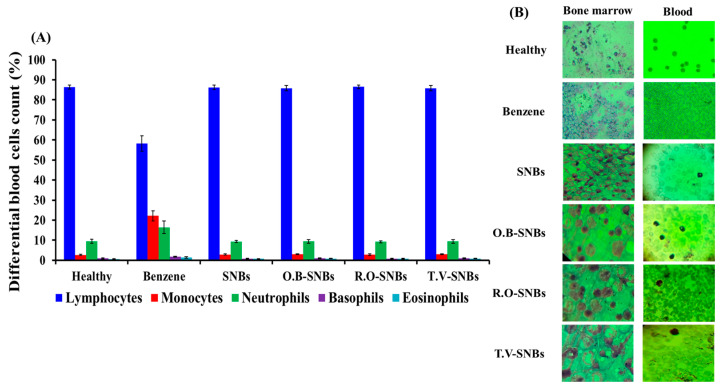
(**A**) Data of differential count of WBCs of healthy rats, those treated with benzene, those treated with only SNBs, those treated with *O. basilicum* plant extract loaded on SNBs (O.B-SNBs), those treated with *R. officinalis* plant extract loaded on SNBs (R.O-SNBs), and those treated with *T. vulgaris* plant extract loaded on SNBs (T.V-SNBs). (**B**) Microscopic examination of bone marrow and blood cell morphology of healthy rats, those treated with benzene, those treated with only SNBs, those treated with *O. basilicum* plant extract loaded on SNBs (O.B-SNBs), those treated with *R. officinalis* plant extract loaded on SNBs (R.O-SNBs), and those treated with *T. vulgaris* plant extract loaded on SNBs (T.V-SNBs).

The average distribution of WBCs in healthy rats consisted of 6% neutrophils, 67% lymphocytes, 8% monocytes, 0.8% eosinophils, and 2.9% basophils. However, in leukaemia-induced rats, the blood cell count was determined weekly. In the first week, an increase in neutrophil count and a decrease in lymphocyte count were observed, resulting in 14% neutrophils, 57% lymphocytes, 8% monocytes, and 1% eosinophils. By the end of the second week, a significant increase in neutrophils (40%) and a decrease in lymphocytes (38%) was recorded, while monocytes decreased by 4%. The third week data is not shown as most of the cells were burst or were damaged due to the benzene action (Figure 5A). In contrast to the dramatic changes observed in blood cell count in benzene-injected rats, rats treated with plant-extract-loaded SNBs displayed a normalized count of blood cells. Specifically, rats treated with *O. basilicum*-loaded SNBs showed the following distribution: 9% neutrophils, 35% lymphocytes, 4% monocytes, 2% eosinophils, and 2.5% basophils. Alternatively, rats treated with *R. officinalis* and *T. vulgaris-*loaded SNBs exhibited a decrease in neutrophil count. These results are shown in Figure 5A. 

### 3.7. Microscopic Examination of Blood and Bone Marrow Cells

All three groups of plant-extracts-loaded-SNBs–treated rats showed very positive results, as both RBCs and WBCs were found to have a regular shape and intact morphology. This was in comparison to the benzene-injected rats shown in Figure 5B. The analysis of the bone marrow revealed a difference in blast cell count among the control group, the benzene-treated (i.e., leukemic) group, and the plant-extract-loaded-SNBs–treated groups. The bone marrow of the normal rats showed erythrocytes (Er), myeloid cells (M), non-segmented neutrophils (NSN), segmented neutrophils (SN), pro-myelocytes (P), and myeloblasts (MB). However, in leukaemia-treated rats, myeloblasts (MB), pro-myelocyte (P), late myelocytes (M), non-segmented neutrophil (NSN), segmented neutrophils (SN), and erythroid precursors (Er) were seen. A large number of blast cells and mature lymphocytes were observed in a leukemic rat. 

Similar results were observed in bone marrow as in blood smears of rats treated with SNBs loaded with the three selected plant extracts. WBC precursor cells, along with erythrocyte precursor cells and a smaller number of blast cells, were also observed. In Giemsa-stained blood smears of leukaemia-induced rats, erythrophagocytosis was observed, where the WBCs engulfed the erythrocytes. There were also burst cells with different shapes, which contrasted with the regularly shaped intact cells seen in smears of healthy rats (Figure 5B).

## 4. Discussion

Nanomedicine has provided new opportunities for enhancing the therapeutic and pharmacological effects of traditional drugs. Nanoparticles, because of their small size and large surface area, have proven to be advantageous in targeted drug delivery [36,37,38,39,40]. By reaching the cellular level and crossing the blood–brain barrier, nanoparticles can deliver drugs specifically to pathological sites, minimizing damage to normal tissues. 

SNBs have a high drug-loading capacity and demonstrate better compatibility both *in vitro* and *in vivo*, with the ability to be safely excreted from the body. XRD, UV–Vis spectroscopy, and SEM were used for the characterization of the prepared SNBs, while FTIR was employed for confirmation of the final formulation. It has been established that nanoparticles less than 50 nm in size, loaded with antitumor drugs, exhibited target specificity [41]. XRD and SEM analysis of the SNBs confirmed a size of approximately 47.9 nm, consistent with other studies [42,43], as well as with that calculated with ImageJ software in this study. The presence of O-H, indicated in FTIR spectra, suggests the presence of phenolic compounds in the extract, which act as antioxidant and anticancer agents. The stretching vibrations of sp^3^ C–H bonds, corresponding to the presence of CH_2_ and CH_3_ groups, indicate the presence of aldehydes and terpenes. FTIR data, specifically peaks at 1076 cm^−1^ and 3162 cm^−1^, confirm the characteristic bonding of Si-O and silanol O-H stretching, which are unique to SNBs, as found in other published data [44]. The FTIR characteristic peaks further validate the presence of various functional groups in phenolics, alkaloids, and other aromatic compounds, which play an effective biological role in combating various diseases [45,46].

The quantity of extract loaded on the SNBs was set as 150 mg/kg body weight of the rat, which is equivalent to 20 mg/kg of body weight of the rats. A previous study has shown that a dosage of 50 mg/kg body weight of silica nanoparticles is sufficient for cancer therapy *in vivo* and *in vitro* [47]. Another study showed that silica nanoparticles decomposed into soluble ortho-silicic acid and were purged through the renal system of the body within four weeks. When administered intravenously, these particles accumulate in the liver, kidney, and spleen. The study further confirmed the safe excretion through the renal route, even if they do not reach the target site [48]. The SNBs showed no significant toxicity to the organs, and their growth was comparable to the control group (data now shown).

The loading of the extract on SNBs was confirmed through UV–Vis spectrophotometric data analysis and the FTIR spectra. The final formulation of the SNBs exhibited characteristic peaks of SiO_2_ and the loaded extract, confirming the presence of basil and CMC on the SNBs. FTIR data also confirmed the successful coating of CMC on the surface of the SNBs, which is consistent with another study that has reported the coating of silica nanoparticles with starch [49]. The coated CMC used in this study was biocompatible and delayed the abrupt release of the drug from the SNBs. This sustained release strategy was employed to ensure that a maximum amount of drugs could reach the target cells while causing minimal damage to normal cells.

To investigate the role of medicinal and nutritional plants in mitigating the haematotoxic effects of benzene metabolites, this study was conducted on Sprague Dawley rats. The rats were injected with benzene, which is known to potentially cause haematotoxicity through mechanisms involving metabolites such as phenol, catechol, hydroquinone, and 1,2,4-benzenetriol [34,35]. Examination of blood smears from all groups of rats indicated severe haematotoxicity due to the presence of band neutrophils and increased monocyte and neutrophil count. Furthermore, the weight of the liver and spleen significantly increased in benzene-injected rats. However, when these rats were concurrently treated with SNBs loaded with extracts of *O. basillicum*, *R. officinalis*, and *T. vulgaris*, the toxic effects induced by benzene were significantly reduced. The weight of the liver and spleen in rats treated with SNBs was nearly normal compared to those treated with benzene alone. Additionally, the organ tissues of these rats appeared morphologically normal (Figure 4A–D), indicating the protective effects of the plant-extract-loaded SNBs against benzene-induced harm in the liver and spleen. Similarly, differential counting of blood cells in benzene-treated rats revealed a significant decrease in lymphocytes, neutrophils, and monocyte count. However, in rats treated with benzene and SNBs loaded with different plant extracts, the cell counts were nearly normal. Morphological examination of blood and bone marrow cells also confirmed that the adverse effects induced by benzene were prevented by these SNBs loaded with plant extracts.

The plant-extract-loaded SNBs were evaluated for their potential to symptomatically improve AML. When rats were treated with benzene (a potent inducer of AML) for two weeks, their blood smear showed an increased leukocyte count, along with neutrophilia, lymphopenia, and a decrease in monocyte levels, confirming AML. It was discovered that lymphopenia during AML may be caused by elevated levels of plasma arginase-II, which stops T-cell proliferation. Additionally, blast cells in the bone marrow shift surrounding monocytes to an immunosuppressive M-2-like state that is also dependent on arginase [50]. This leads to a low monocyte count. In comparison to the control bone marrow, leukemic rats had a higher number of blast cells in their bone marrow. 

After three weeks of benzene administration, leukocytes were disrupted and distorted, and the bone marrow had a higher number of blast cells. Treatment after three weeks did not show significant improvement, likely due to the severity of the disease. Therefore, treatment began after the second week of AML induction, which resulted in improvement of the AML condition. The differential count after treatment with plant-extract-loaded SNBs showed a significant decrease in the neutrophil count, bringing it back to normal levels. The lymphocyte count returned to the normal range, and the monocyte count decreased. Our findings align with a previous study, which demonstrated that *O. basilicum* has significant activity against benzene-induced haematotoxicity in mice [26]. The bone marrow of benzene-treated rats had a higher number of blast cells compared to the bone marrow of the control and extract-loaded SNBs-treated rats. However, more information is needed about the bone marrow niches involved in haematopoiesis and their response to benzene [51]. In conclusion, generic SNBs have physical properties that make them suitable as drug carriers. The plant-extract-loaded SNBs have the potential to minimize the impact of benzene on AML, as demonstrated by the differential count of WBC, analysis of organ weight, morphological analysis, and bone marrow analysis. 

LC-MS analysis of the selected fraction of various plant extracts revealed the presence of flavonoids, ester derivatives of amino acids, and carboxylic derivatives. The presence of these compounds strongly suggests that these fractions have a role in fighting leukaemia. Studies have reported that epicatechin and caffeic acid derivatives have anticancer properties, with epicatechin oligomers suppressing the expression of the cancer-promoting gene. (−)-Epicatechin is an aromatic compound known for its ability to scavenge oxygen and modulate cell signalling in the MAP kinase pathway [52,53,54]. Additionally, amino acid ester derivatives have been found to have anti-leukemic properties [55]. Cinnamic acid derivatives, which have aromatic rings and a polyphenolic structure, have also been used as anticancer agents [56]. Similarly, the presence of carboxylic acid derivatives further strengthens the potential of anticancer activity of the selected fractions. In this study, both LC-MS and FTIR data support each other, indicating the need for further investigation to understand the mechanistic pathway of AML induction. Furthermore, the impact of prescribed plant extracts as nanomedicine could be explored through the expression of arginase-II, TNF-α, and relevant pro-inflammatory cytokines.

## 5. Conclusions

In summary, this study highlights the potential of using silica nanobeads (SNBs) loaded with methanolic extracts from *O. basilicum*, *R. officinalis*, and *T. vulgaris* as a promising approach to mitigate the haematotoxic effects caused by exposure to benzene. The successful preparation and characterization of SNBs, with an average size of approximately 48 nm, along with the confirmation of loading of plant extracts through UV–Vis and FTIR analyses, indicate that this nanotheragnostic method is feasible. Intravenous administration of the final nanomedicine to benzene-exposed rats yielded significant improvements in haematological parameters, notably reducing the count of WBCs and eliminating blast cells. These findings suggest that SNBs can effectively deliver medicinal herbs in very low doses, making them excellent targeted drug delivery agents. The potential of this approach offers promise in addressing the haematotoxic effects caused by benzene exposure and provides new opportunities for developing safer and more effective treatments for individuals at risk of benzene-related health problems. Further research and clinical investigations are warranted to fully explore the therapeutic potential of SNBs in mitigating the toxic effects of benzene and enhancing the overall quality of life for affected individuals.

## Figures and Tables

**Table 1:** LC-MS results of the selected fractions RF1, RF2, TF2, TF7, and TF8.

Fraction	Compounds	MS (*m*/*z*)	Other Fragment Ions	UV Maxima (nm)
RF1	(-) Epicatechin derivative	922.1	707.3, 365.6, 187.6, 129.1	240, 320
	Caffeic acid-3-glucoside (Dimer)	683.1	341.5	242, 330
RF2	D-Alanine, N-(2,5-ditrifluoromethylbenzoyl), nonadecyl ester	545.0	507.2, 425.9	280, 330
	Rosemarinic acid	553.2	187.8	265, 330
TF2	Trans-3-trifluoromethyl cinamic acid, 3-4-dichlorophenyl ester (Dimer)	719.4	360	230, 322
	Thymol	756.7	361.4, 856.7	280, 322
TF7	Kaempferol-3-o-alpha-l-rhamopyranosyl-(1-6)-beta-d-galactopyranosyl]7-o-alpha-l-rhamanopyranoside (flavonoid)	945.1	470.5, 407.5	262
	Carvecrol	956.0	391.1, 203.8	260, 290, 330
	Succinic acid, 3,5-dichlorophenyl hexadecyl ester	956.2	486.5, 263.0	230, 280
TF8	Trans-3-trifluoromethyl cinamic acid, 3-4-dichlorophenyl ester (Dimer)	719.4	360	220, 320
	Trans-3-trifluoromethyl cinamic acid, 3-4-dichlorophenyl ester derivative	756.7	361.4, 856.7	270, 325

## Data Availability

No new data were created or analysed in this study. Data sharing is not applicable to this article.

## Supplementary Materials

The following supporting information can be downloaded at: https://www.mdpi.com/article/10.3390/toxics11100865/s1, Figure S1: MS spectra of different fractions of extracts; Figure S2: LC-DAD chromatograms of different fractions of methanolic extracts.
