# Supplementary material for: Mitigation of Benzene-Induced Haematotoxicity in Sprague Dawley Rats through Plant-Extract-Loaded Silica Nanobeads
Source: Toxics. 2023 Oct 17;11(10):865. doi: 10.3390/toxics11100865 (PMC10610980; doi:10.3390/toxics11100865)
Supplement: Supplementary file 1 [file toxics-11-00865-s001.zip › toxics-2620110-supplementary.pdf]

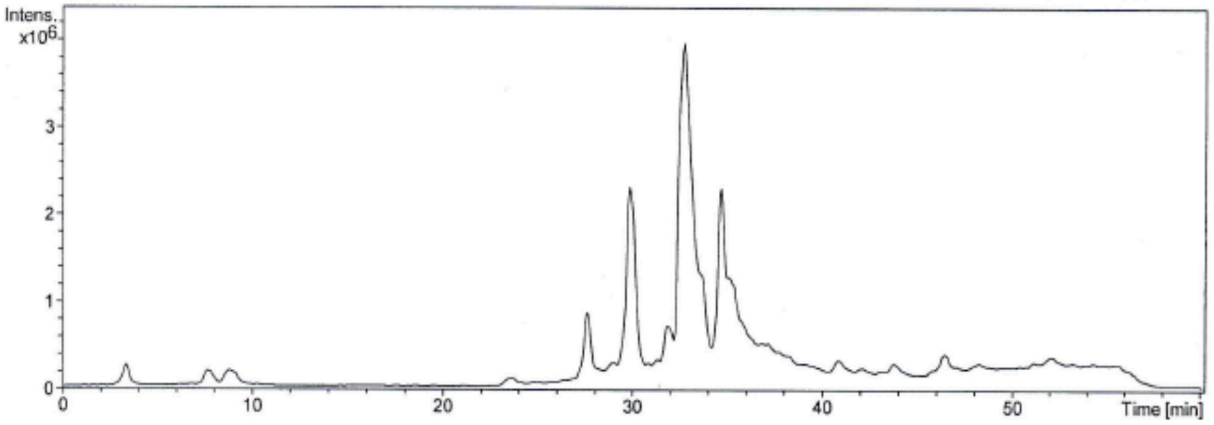
Intens.
x10^6
0
1
2
3
10
20
30
40
50
Time [min]

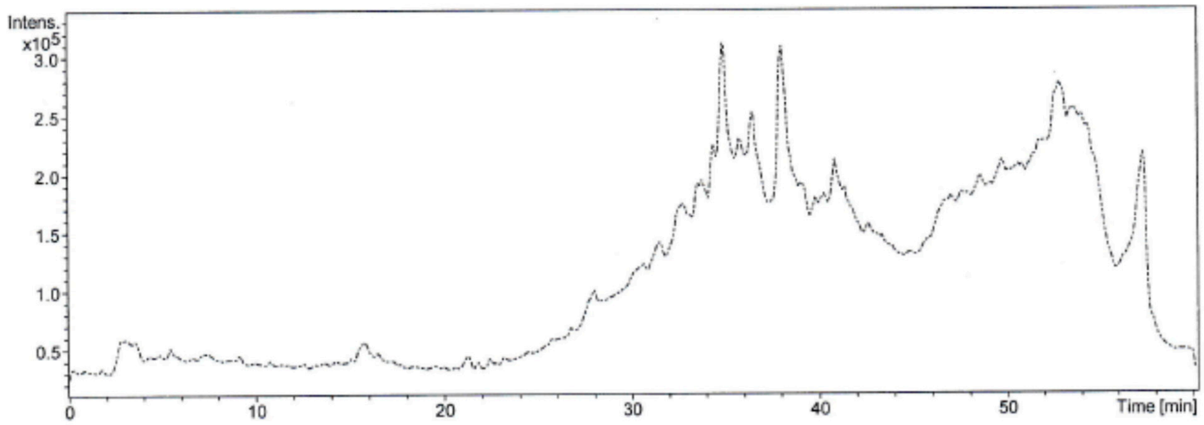
Intens.
x10^5
0.5
1.0
1.5
2.0
2.5
3.0
0
10
20
30
40
50
Time [min]

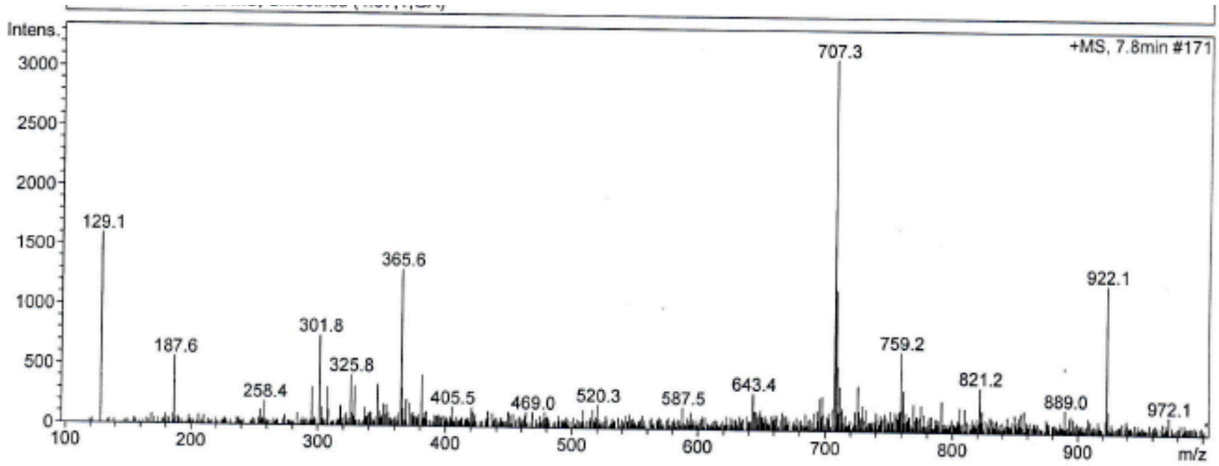
Intens.
+MS, 7.8min #171
0
500
1000
1500
2000
2500
3000
129.1
187.6
258.4
301.8
325.8
365.6
405.5
469.0
520.3
587.5
643.4
707.3
759.2
821.2
889.0
922.1
972.1
100
200
300
400
500
600
700
800
900
m/z

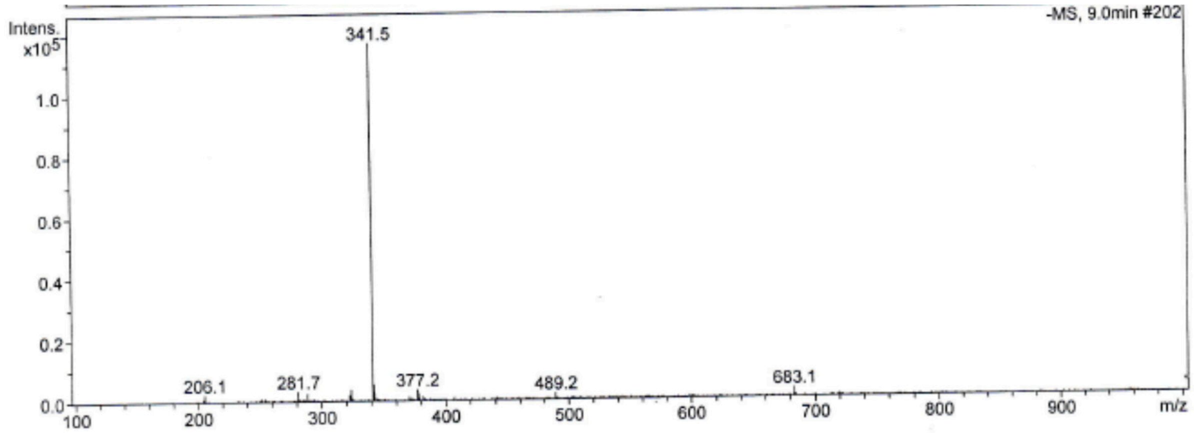
Intens.
x10^5
-MS, 9.0min #202
341.5
206.1
281.7
377.2
489.2
683.1
m/z

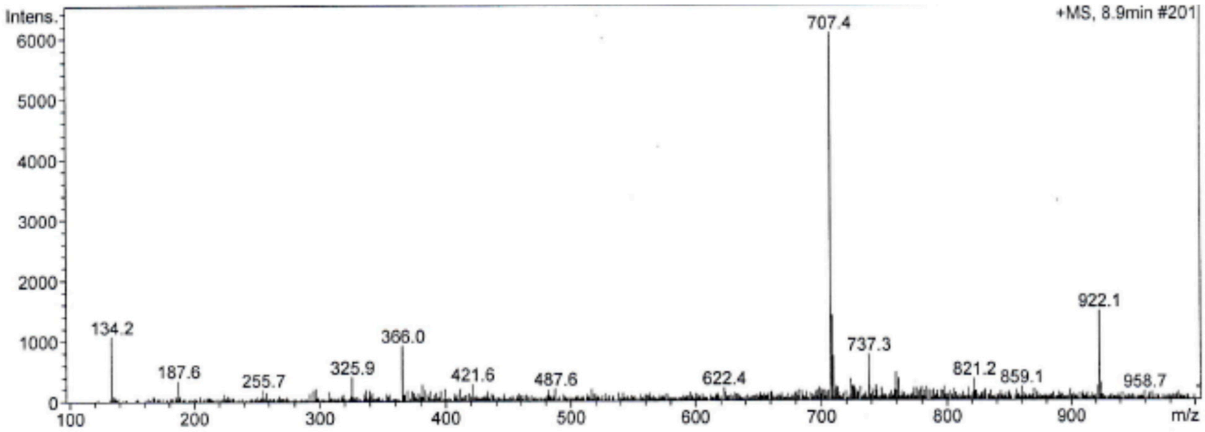
Intens.
+MS, 8.9min #201
707.4
922.1
134.2
366.0
737.3
187.6
325.9
421.6
255.7
487.6
622.4
821.2
859.1
958.7
m/z

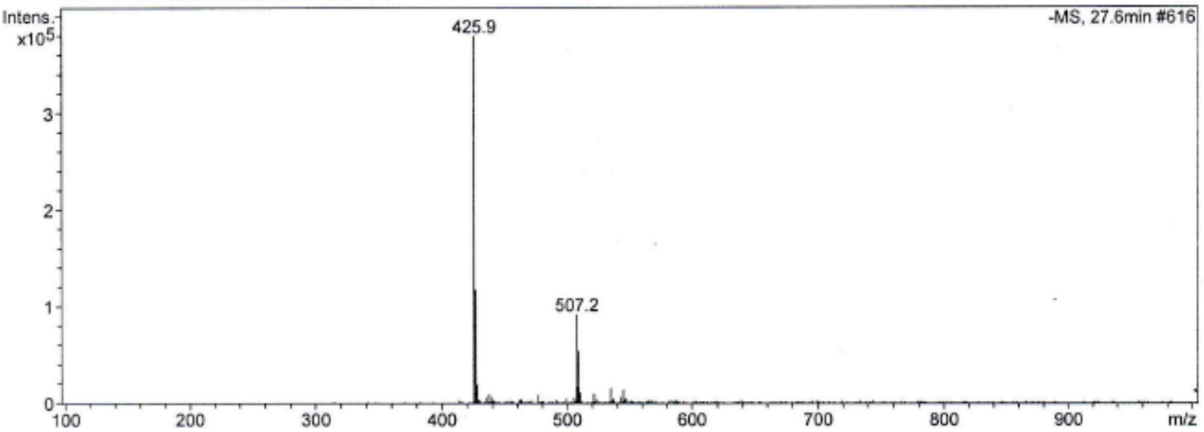
Intens.
x10^5
-MS, 27.6min #616
425.9
507.2
m/z

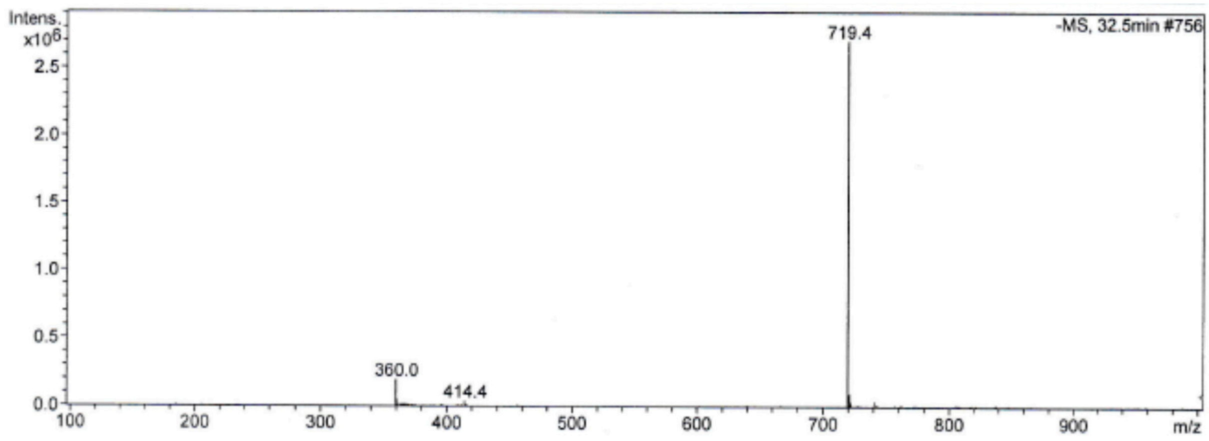
Intens.
x10^6
-MS, 32.5min #756
719.4
360.0
414.4
m/z

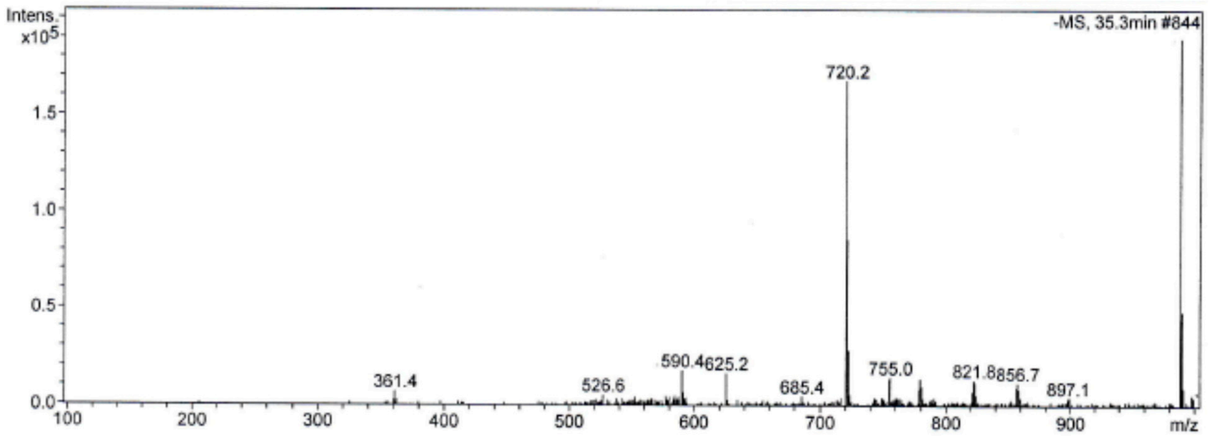
Intens.
x10^5
-MS, 35.3min #844
720.2
361.4
526.6
590.4
625.2
685.4
755.0
821.8
856.7
897.1
m/z

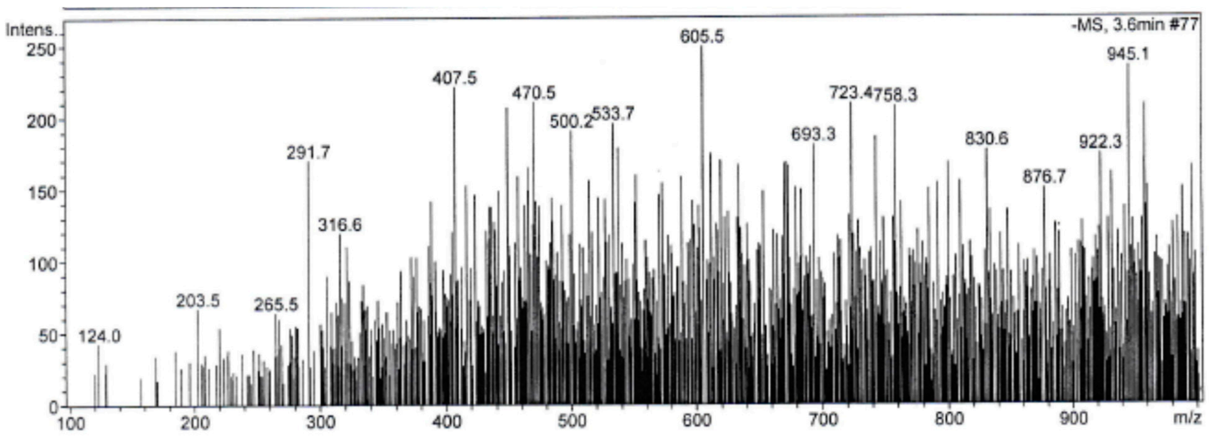
Intens.
-MS, 3.6min #77
605.5
945.1
407.5
470.5
500.2
533.7
723.4
758.3
693.3
830.6
922.3
876.7
291.7
316.6
203.5
265.5
124.0
m/z

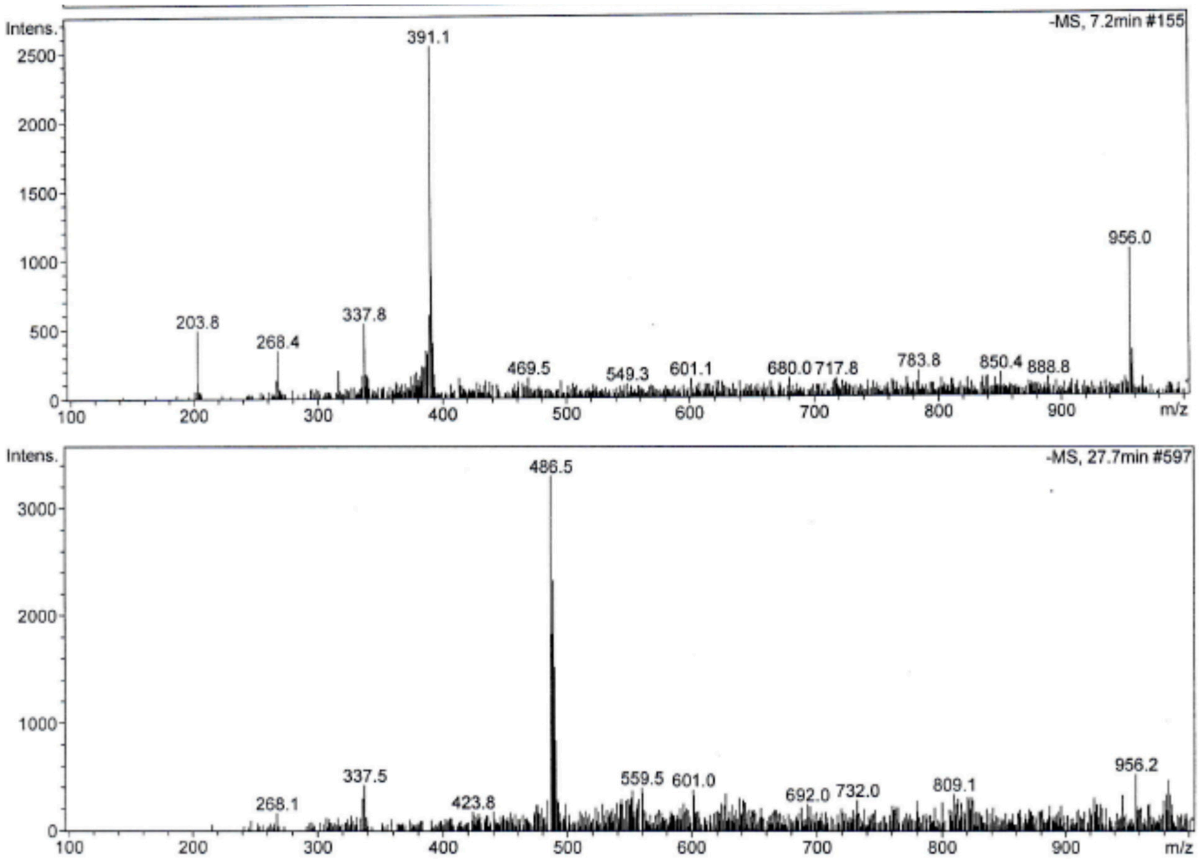

Figure S1. MS spectra of different fractions of extracts.

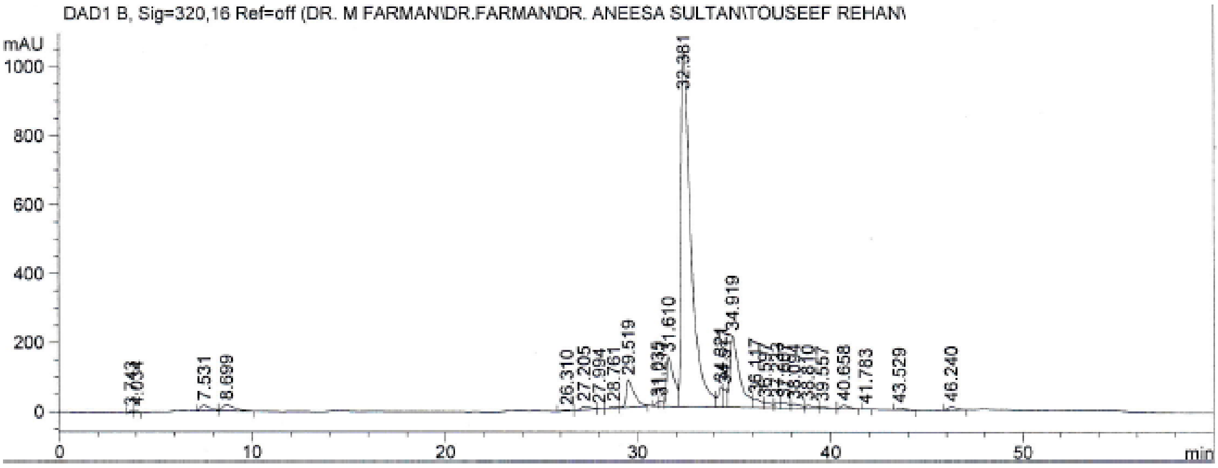

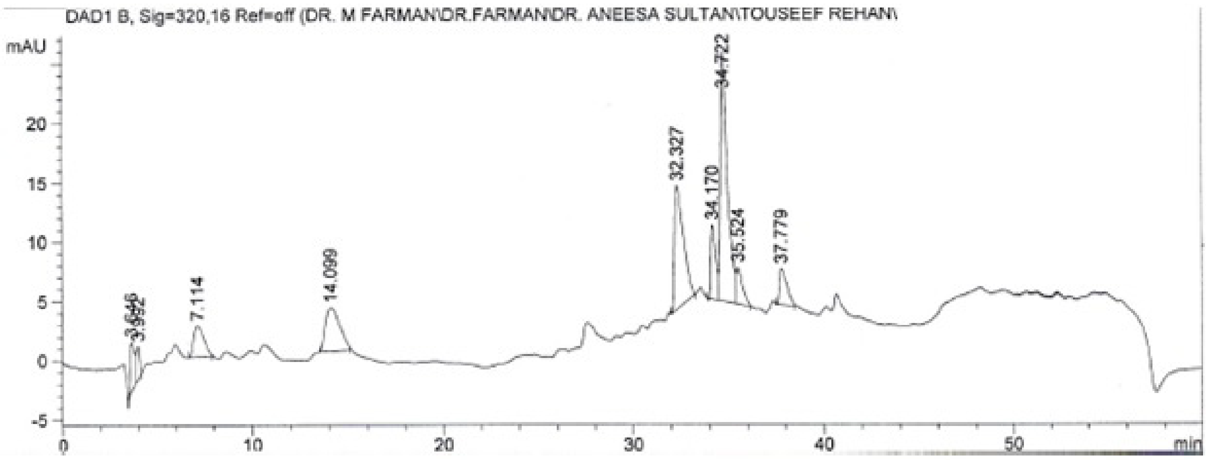
DAD1 B, Sig=320,16 Ref=off (DR. M FARMAN\DR.FARMAN\DR. ANEESA SULTAN\TOUSEEF REHAN\
mAU
min
7.114
14.099
32.327
34.170
34.722
35.524
37.779
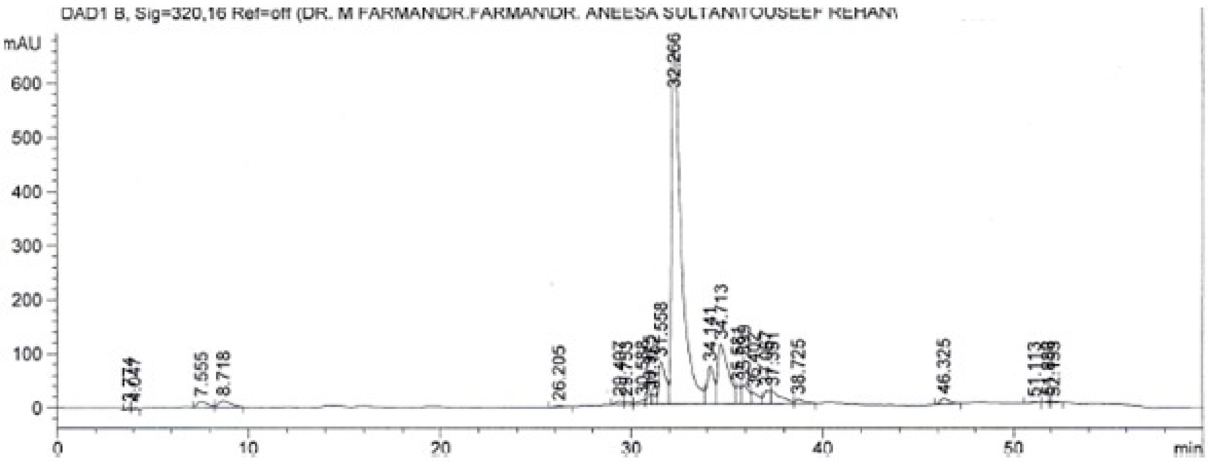
DAD1 B, Sig=320,16 Ref=off (DR. M FARMAN\DR.FARMAN\DR. ANEESA SULTAN\TOUSEEF REHAN\
mAU
min
7.555
8.718
26.205
31.558
32.266
34.141
34.713
38.725
46.325
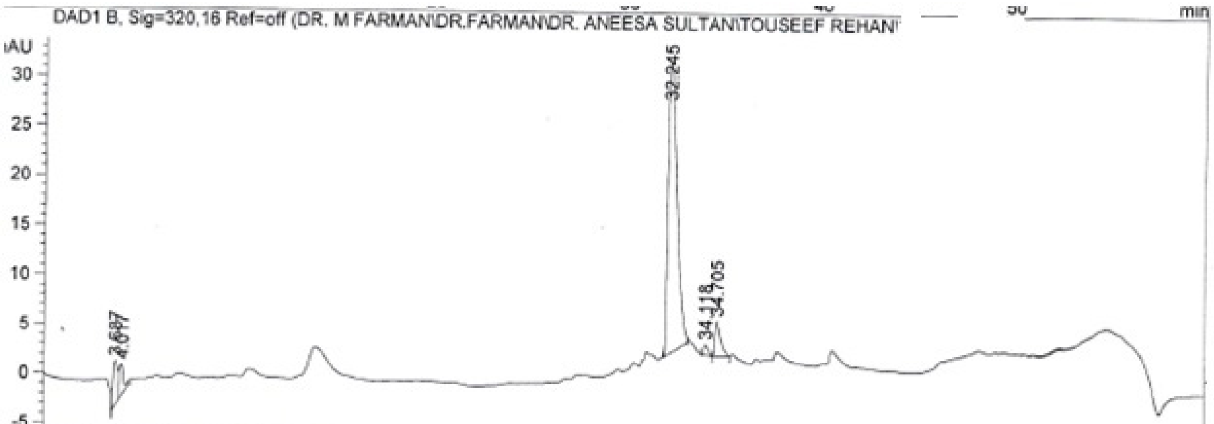
DAD1 B, Sig=320,16 Ref=off (DR. M FARMAN\DR.FARMAN\DR. ANEESA SULTAN\TOUSEEF REHAN\
min
32.245
34.118
34.705

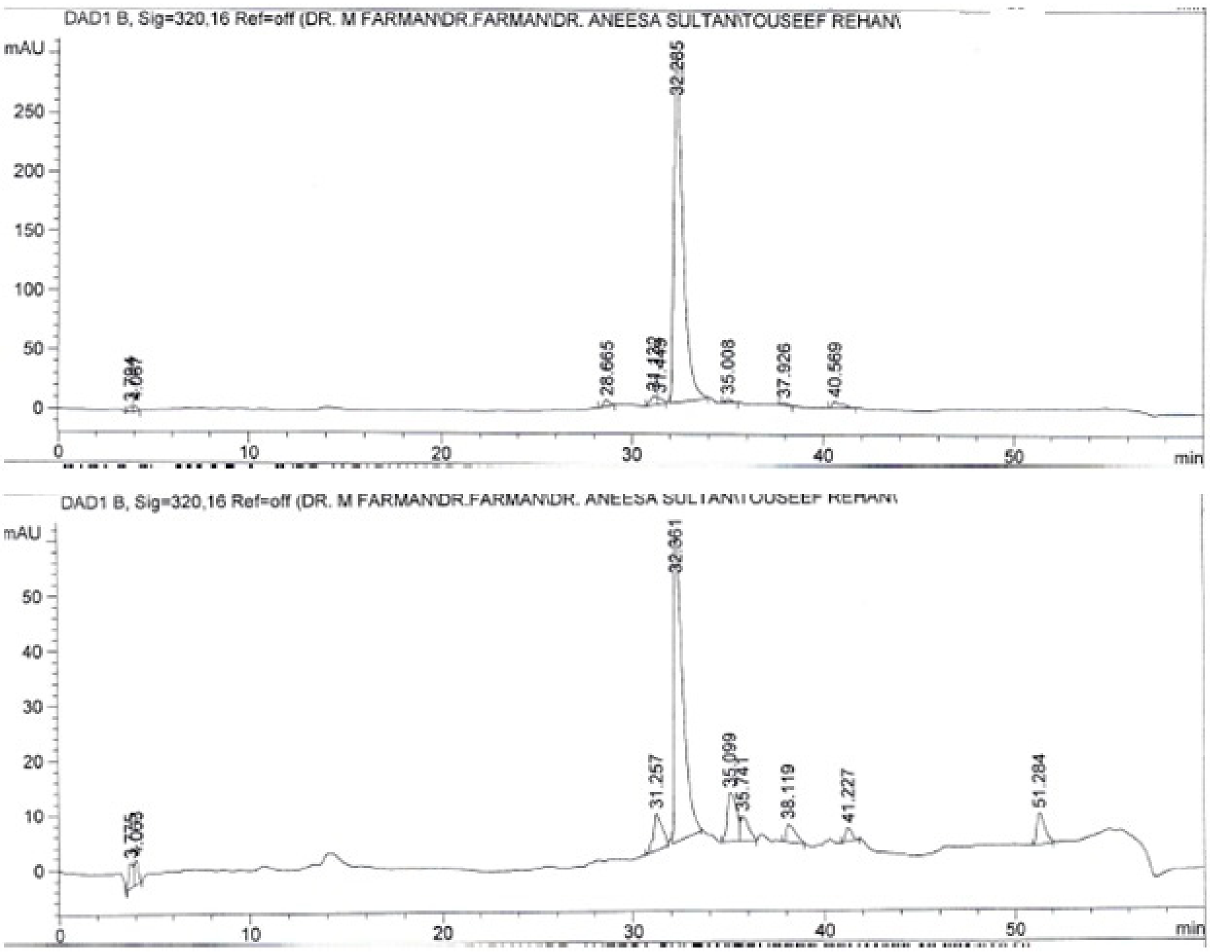

Figure S2. LC-DAD chromatograms of different fractions of methanolic extracts.
